# Effects of Interval Training Under Hypoxia on Hematological Parameters, Hemodynamic Function, and Endurance Exercise Performance in Amateur Female Runners in Korea

**DOI:** 10.3389/fphys.2022.919008

**Published:** 2022-05-18

**Authors:** Hun-Young Park, Won-Sang Jung, Sung-Woo Kim, Jisu Kim, Kiwon Lim

**Affiliations:** ^1^ Department of Sports Medicine and Science, Graduate School, Konkuk University, Seoul, South Korea; ^2^ Physical Activity and Performance Institute (PAPI), Konkuk University, Seoul, South Korea; ^3^ Department of Physical Education, Konkuk University, Seoul, South Korea

**Keywords:** hypoxia, interval training, immune function, hemodynamic function, endurance exercise performance, amateur female runners

## Abstract

Interval training under hypoxia (IHT) is commonly used to enhance endurance exercise performance. However, previous studies examining hematologic changes related to the immune system that affect health and conditioning are lacking. This study aimed to evaluate the effects of IHT for 6-weeks on hematological parameters, hemodynamic function, and endurance exercise performance in amateur Korean female runners. Twenty healthy amateur Korean female runners (age: 24.85 ± 3.84 years) were equally assigned to normoxic training group (NTG) for interval training under normoxia (760 mmHg) and hypoxic training group (HTG) for interval training under hypobaric hypoxia (526 mmHg, 3000 m simulated altitude) according to their body composition and endurance exercise performance. All participants performed 120-min of training sessions, consisting of 20-min of warm-up, 60-min of interval training, and 20-min of cool-down. The training program was performed 3-days per week for 6-weeks. Warm-up and cool-down were performed for 20-min at 60% maximal heart rate (HRmax). The interval training sessions comprised 10 repetitions of interval exercise (5-min of exercise corresponding to 90–95% HRmax and 1-min of rest) on a treadmill. All participants underwent measurements of hematological parameters, hemodynamic function, and endurance exercise performance before and after training. Both groups showed a significant increase in erythropoietin (EPO) level and a decrease in monocyte abundance, with EPO showing a greater increase in the HTG than in the NTG. B cell abundance significantly increased in the NTG; hematocrit and neutrophil counts significantly increased, and lymphocyte counts significantly decreased in the HTG. The HTG showed a significant improvement in oxygen uptake, stroke volume index, and end-diastolic volume index compared to the NTG. In addition, both groups showed significant improvements in heart rate, end-systolic volume index, and cardiac output index. The maximal oxygen uptake and 3000 m time trial record were significantly improved in both groups, and the HTG showed a tendency to improve more than the NTG. In conclusion, the IHT was effective in enhancing endurance exercise performance through improved hemodynamic function. Furthermore, hematological parameters of immune system showed a normal range before and after training and were not negatively affected.

## Introduction

Hypoxic training is often used to improve endurance exercise performance; however, a previous study reported that adding hypoxia as an additional stressor led to no additional benefit in 50% of studies (Millet et al., 2010). Faiss et al. and McLean et al. confirmed that the enhancement in endurance exercise performance following hypoxic training was more strongly related to exercise training performed using the high-intensity anaerobic interval method (Faiss et al., 2013; McLean et al., 2014). Therefore, the exercise protocol for improving endurance exercise performance mainly utilizes high-intensity interval exercises rather than moderate-intensity continuous exercises (McLean et al., 2014; Sinex and Chapman, 2015; Czuba et al., 2017; Park et al., 2018; Jung et al., 2020).

Among the various hypoxic training methods, the interval training under hypoxia (IHT) protocol is composed of repeated exposure to 5–7 min of steady or progressive hypoxia during a high-intensity training session, interrupted by equal periods of recovery (Bernardi, 2001; Jung et al., 2020). IHT may enhance erythropoietic, metabolic, and hemodynamic functions, which stimulate serum erythropoietin synthesis, elevate red blood cell volume, improve exercise economy, and increase blood flow, leading to enhanced blood supply and utilization of tissues (Geiser et al., 2001; Hamlin et al., 2010; Czuba et al., 2011). Additionally, it is known to enhance endurance exercise performance by improving capillary and mitochondrial density, oxidative enzyme activity, metabolic functions, such as anaerobic glycolysis and lactate tolerance, hemodynamic function, vascular endothelial growth factor, and nitric oxide levels (Geiser et al., 2001; Hamlin et al., 2010; Czuba et al., 2017; Park and Lim, 2017; Żebrowska et al., 2019; Chacón Torrealba et al., 2020; Jung et al., 2020). However, the enhancement of endurance exercise performance following IHT under normoxia is controversial. Furthermore, a few previous studies have not supported the enhancement of endurance exercise performance following IHT (Katayama et al., 2004; Rodríguez et al., 2007; Roels et al., 2007; Beidleman et al., 2009). These conflicting results may be due to methodological differences, such as athletes’ training status; type, volume, and intensity of training; hypoxic stimulus dose; and time point in the measurement of athletic performance following the hypoxic training procedure (McLean et al., 2014; Sinex and Chapman, 2015; Park et al., 2018). These previous studies used a relatively low exercise intensity during training and short exposure periods under hypoxia. In other words, IHT should consist of an appropriate training modality with a high-intensity and anaerobic interval method to maximize its effectiveness (McLean et al., 2014).

Exercise training-induced stress under hypoxia changes physiological and metabolic functions more than that under normoxia (Mazzeo et al., 2001), affecting the nervous and endocrine systems, as well as the immune system (Mazzeo, 2008). Hypoxic exposure stimulates the release of catecholamines, such as epinephrine and norepinephrine, in the adrenal medulla, activates the sympathetic nervous system (SNS), and increases the concentration of cortisol and corticosteroids in plasma (Mazzeo et al., 2000; Beidleman et al., 2006). The most notable changes in immune system following hypoxic exposure include a decreased lymphocyte count, T cell count, and T cell activation and proliferation, and increased upregulation of inflammatory cytokines, such as interleukin (IL)-1, IL-6, C-reactive protein (CRP), and tumor necrosis factor (TNF)-α by hypoxic inducible factors (Hartmann et al., 2000; Pedersen and Steensberg, 2002; Thake et al., 2004; Mazzeo, 2008; Jung et al., 2020; Chen and Gaber, 2021). In particular, the elevation of IL-6 following hypoxic exposure is indicated by an increase in epinephrine release due to an increase in the activity of the *ß*-adrenergic pathway in acute conditions and by an increase in the release of norepinephrine due to an increase in the activity of the *a*-adrenergic pathway in chronic conditions (van Gool et al., 1990; Mazzeo et al., 2001). Elevated IL-6 levels lead to enhanced blood supply and utilization to tissues *via* expression of vascular endothelial growth factor (VEGF) mRNA, increase in erythropoietin (EPO) level, and improved reticulocyte production (Faquin et al., 1992; Cohen et al., 1996). As mentioned above, hypoxic exposure induces hematological changes related to immune system, revealed by various changes in the physiological, metabolic, and neuroendocrine systems. However, there are few previous studies that have verified the changes of hematological parameters including oxygen transporting capacity and immune system following exercise training under hypoxia.

Hypoxic training may enhance endurance exercise performance under normoxia due to hematological and non-hematological changes; therefore, it is important to investigate the effect on hematological parameters including immune function regarding health and conditioning (Camacho-Cardenosa et al., 2017; Jung et al., 2020; Camacho-Cardenosa et al., 2021). Moreover, the World Anti-Doping Agency is concerned that exercise training regimens under hypoxic conditions can have a potentially negative effect on health (Wilber, 2007). For the general use of hypoxic training in various participants (e.g., elite athletes, healthy participants, women, the elderly, and adolescents), it is essential to investigate the effects of exercise training under hypoxia on hematological parameters, including oxygen transport capacity and immune system, and establish the efficacy and safety of hypoxic training.

Therefore, the present study aimed to investigate the effects of 6-weeks interval training under moderate hypobaric hypoxia (526 mmHg, 3000 m simulated altitude) and normoxia (760 mmHg) on hematological parameters, hemodynamic function, and endurance exercise performance in amateur Korean female runners. We hypothesized that IHT would enhance endurance exercise performance because of hematological and non-hematologic changes and would not adversely affect hematological parameters related to immune system in these runners.

## Materials and Methods

### Participants

The present study included 20 amateur Korean female runners (age: 19–29 [24.85 ± 3.84] years) who has continuously participated in track and field competitions at least 4 times a year for at least 5-years. An a priori power analysis was performed with G-power 3.0 for the endurance exercise performance (VO_2_max and 3000 m time trial) based on previous research (Jung et al., 2020), indicating that a sample size of 16 participants (8 participants per group) would be required to provide 85% power at an *a*-level of.05. We anticipated a more than 10% dropout rate and aimed for a starting population of 20. The criteria for exclusion of participants are as follows: exposure to hypoxia in the 6 months prior to the present study; intake of any medication or nutritional supplements during the 3 months prior the present study; smoking; any kind of hypertension, diabetes, hyperlipidemia, or cardiovascular, pulmonary, or musculoskeletal diseases.

They were equally assigned to a normoxic training group (NTG) for interval training under normoxia (760 mmHg) or a hypoxic training group (HTG) for interval training under hypoxia (526 mmHg, simulated altitude of 3000 m) according to their body composition and endurance exercise performance. All participants received information about the purpose and process of the study, including possible side effects, and consent was obtained. All participants completed the study; thus, all data were used in the analyses. As shown in Table 1, there were no significant differences in physical characteristics between the groups. All procedures were in accordance with the ethical standards of the responsible committee on human experimentation and with the Declaration of Helsinki. The study was approved by the Institutional Review Board of Konkuk University (7001355–2020002-HR-359) in Korea and was conducted according to the Declaration of Helsinki.

**TABLE 1:** Participants’ characteristics.

Variables	NTG	HTG	*t*-value	*p*-value
Number (n)	*n* = 10	*n* = 10	—	—
Environmental condition (mmHg)	Sea level (760 mmHg)	3000-m simulated altitude (526 mmHg)	—	—
Age (year)	24.5 ± 3.8	25.2 ± 4.0	-0.398	0.695
Height (cm)	164.8 ± 0.9	163.2 ± 3.9	1.243	0.243
Weight (kg)	52.0 ± 3.4	51.2 ± 4.7	0.409	0.687
BMI (kg/m^2^)	19.1 ± 1.2	19.2 ± 1.6	-0.140	0.890
FFM (kg)	35.6 ± 2.3	35.5 ± 3.2	0.057	0.955
Percent body fat (%)	20.7 ± 0.9	20.6 ± 1.1	0.090	0.929

Note. Values are expressed as mean ± standard deviation. NTG, normoxic training group; HTG, hypoxic training group; BMI, body mass index; FFM, free fat mass.

### Study Design

**FIGURE 1:**
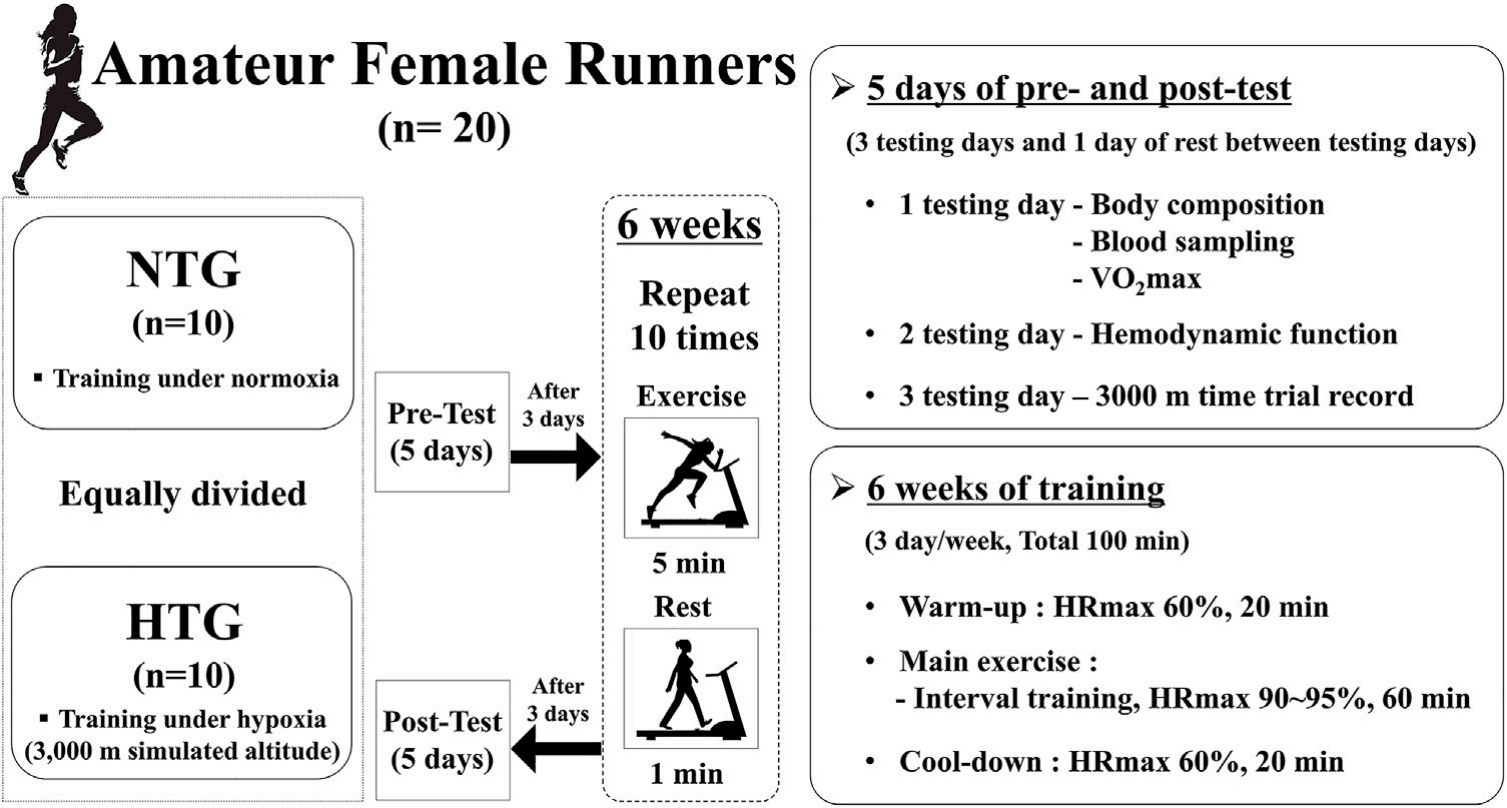
Study design. Difference in hemorheological function (mean ± SD) between non-obese and obese. Note. NTG = normoxic training group, HTG = hypoxic training group, HRmax = maximal heart rate, VO_2_max = maximal oxygen uptake.

The present study design is illustrated in Figure 1 and was similar to that of Jung et al. (2020). Our study design included a 5-days pre-test period (i.e., 3 testing days and 2 rest days between testing days) under normoxia (e.g., 3 testing days and 1 resting day between testing days), a 6-weeks interval training session period under each environmental condition (normoxia or hypoxia), and a 5-days post-test period (e.g., 3 testing days and 1 resting day between testing days).

The pre-test period was completed before the start of 5-days training, and the post-test period began 5 days after the last training session. On the first test day, all participants fasted for >8 h, and body composition was measured after stabilization. Thereafter, a venous blood sample was collected to analyze hematological parameters, including red blood cell (RBC) count, hemoglobin (Hb) concentration, hematocrit (Hct), erythropoietin (EPO) concentration, white blood cell (WBC) count, monocytes, eosinophils, neutrophils, basophils, lymphocytes, B cells, T cells, and natural killer (NK) cells, which were measured between 7:00 and 9:00 AM in the resting state. After sufficient rest and meals, maximal oxygen uptake (VO_2_max) and maximal heart rate (HRmax) were measured to evaluate endurance exercise performance in the afternoon. On the second test day, hemodynamic function parameters, including heart rate (HR), oxygen consumption (VO_2_), stroke volume index (SVi), end-diastolic volume index (EDVi), end-systolic volume index (ESVi), and cardiac output index (COi), were measured during a 30 min bout of submaximal cycle ergometer exercise. Exercise intensity during submaximal exercise was set at individual cycle ergometer exercise load values corresponding to 70% VO_2_max obtained during the pretest period. On the third test day, a 3000 m time trial record was recorded in an authorized stadium.

During the interval training session, all participants performed 120 min training sessions consisting of warm-up, interval training, and cool-down under each environmental condition (NTG: normoxic condition, 760 mmHg; HTG: hypobaric hypoxic condition, 526 mmHg, a 3000 m simulated altitude). The training program was performed 3 days per week for 6 weeks. Warm-up and cool-down were performed for 20 min at an intensity of 60% HRmax. The interval training sessions comprised 10 repetitions of interval exercise (5 min of exercise corresponding to 90–95% HRmax and 1 min of rest) on a treadmill. All training sessions were supervised by the investigators.

Before and after the 6-weeks interval training session under each environmental condition, all participants underwent body composition (e.g., weight, body mass index [BMI], free fat mass, and percent body fat), hematological parameter (e.g., RBC count, Hb concentration, Hct, EPO concentration, WBC count, monocytes, eosinophils, neutrophils, basophils, lymphocytes, B cells, T cells, and NK cells), hemodynamic function (HR, VO_2_, SVi, EDVi, ESVi, and COi), and endurance exercise performance (VO_2_max and 3000 m time trial record) assessments.

All testing procedures and training sessions were performed in a 9-m (width) × 7-m (length) × 3-m (high) chamber at a temperature of 22 ± 1°C and humidity of 50 ± 5%, regulated by an environmental control chamber (NCTC-1, Nara control, Seoul, Republic of Korea).

### Measurements

#### Body Composition

Body composition parameters including height, weight, BMI, free fat mass, fat mass, and body fat percentage of all participants were estimated using a stadiometer (YM-1, KDS, Seoul, Korea) and a bioelectrical impedance analyzer (Inbody 770; Inbody, Seoul, Korea). All participants wore lightweight clothing and were asked to remove all metallic items from their bodies.

#### Hematological Parameters

For hematological parameters, 6-ml venous blood samples were obtained before and after the training session at rest under normoxia. A 5-ml venous blood sample was collected in an ethylenediaminetetraacetic acid (EDTA) tube for whole blood, and a 1-ml blood sample was collected in a serum separation tube for serum. The EDTA tube for whole blood was inverted gently several times immediately after venous blood collection to ensure anti-coagulation, and the tube was stored in a refrigerator (−20°C) until the time of the assay. The venous blood sample in the serum separation tube was centrifuged at 3,500 rpm for 10 min. Serum was stored at −70°C until the time of the assay. Thereafter, frozen or refrigerated serum was commissioned by the Clinical Laboratory of Green Cross Medical Foundation and analyzed using the method described below.

RBCs, Hb, Hct, WBCs, neutrophils, eosinophils, and basophils were measured using an XE2100D hematology analyzer (Sysmex, Kobe, Japan) by flow cytometry using a cellpack kit (Sysmex, Kobe, Japan). EPO was analyzed using an Immulite 2000 XPI analyzer (Siemens, Eschborn, Germany) *via* chemiluminescent immunoassay. B, T, and NK cells were analyzed using FC500 (Beckman Coulter, CA, United States) and measured *via* flow cytometry using a CD19-PE kit (Beckman Coulter, Paris, France), CD3-PC5 kit (Beckman Coulter, Paris, France), and NK cell kit (Beckman Coulter, Paris, France), respectively.

All analyses were performed in duplicate, and the average value was used as the measurement value.

#### Hemodynamic Function

Hemodynamic function was measured while the participants performed a 30 min bout of submaximal cycle ergometer exercise corresponding to 70% VO_2_max obtained during the pre-test period under normoxia. In hemodynamic function parameters, VO_2_ was measured using the K5 auto metabolism analyzer (Cosmed, Rome, Italy) and a breathing valve in the form of a facemask. HR, SVi, EDVi, ESVi, and COi were assessed noninvasively using a thoracic bioelectrical impedance device (PhysioFlow PF-05, Paris, France). All parameters were measured every minute, and the total summation value for 30 min was used as the measurement value.

#### Endurance Exercise Performance

Regarding endurance exercise performance, VO_2_max was measured before and after the training sessions with the modified BRUCE protocol for graded exercise test on a treadmill (S25TX, SFET, Seoul, Korea) using a K5 breath-by-breath auto metabolism analyzer (K5, Cosmed, Rome, Italy) and a breathing valve in the form of a facemask. The VO_2_max had the highest VO_2_ obtained during the concluding period of the graded exercise test. The graded exercise test was completed when the following four criteria were satisfied: 1) VO_2_ plateau, no further increase in oxygen use per minute even with an increase in work performed; 2) HR within 10 beats of the age-predicted HRmax; 3) respiratory exchange ratio >1.5; and 4) plasma lactate concentration >7 mmol/L.

The 3000 m time trial record was measured in an authorized stadium under normoxia in Seoul between 8:00 and 10:00 a.m. (temperature = 20–24°C; humidity = 50–60%; wind = 0–10 km/h). To avoid the effect of racing strategies, all running starts were staggered for at least three minutes.

### Statistical Analysis

Means and standard deviations were calculated for all dependent parameters. The normality of the distribution of all outcome parameters was verified using the Shapiro–Wilk W-test prior to parametric tests. A two-way analysis of variance (“time” × “group”) with repeated measures on the “time” factor was used to analyze the effects of training program on each dependent parameter. If a significant interaction was found, a paired *t*-test was performed to compare the post-training and pre-training values of the dependent parameters in each group separately. We used Cohen’s d (effect size), which reflects the value of a statistic calculated from a sample of data and standardized mean differences. Statistical differences in the means (effect size, Cohen’s d) were determined at a significance level of *p* < 0.05 and 95% confidence interval (CI). All analyses were performed using SPSS (version 25.0; IBM Corp., Armonk, NY, United States).

## Results

### Body Composition

The body composition results for the NTG and HTG before and after the training sessions are presented in Table 2. No significant interaction was observed for any of the body composition parameters. Body composition did not affect changes in hematological parameters, hemodynamic function, or endurance exercise performance.

**TABLE 2:** Changes in body composition parameters before and after 6 weeks exercise training in each environmental condition.

Variables	NTG (*n* = 10)		Cohen’s d (95% CI)	HTG (*n* = 10)		Cohen’s d (95% CI)	*p*-value (η^2^)		
	Pre	Post		Pre	Post		Group	Time	Interaction
Weight (kg)	52.0 ± 3.4	51.7 ± 3.3	−0.07 (−0.90, 0.78)	51.2 ± 4.7	51.1 ± 4.4	−0.03 (−0.87, 0.81)	0.697 (0.009)	0.057) (0.187)	0.659 (0.011)
BMI (kg/m^2^)	19.1 ± 1.2	19.0 ± 1.1	−0.07 (−0.91, 0.77)	19.2 ± 1.6	19.2 ± 1.6	0.00 (−0.83, 0.84)	0.829 (0.003)	0.191) (0.093)	0.119 (0.130)
FFM (kg)	35.6 ± 2.3	35.4 ± 2.4	−0.07 (−0.90, 0.78)	35.5 ± 3.2	35.5 ± 3.2	0.00 (−0.84, 0.84)	1.000 (0.000)	0.539) (0.021)	0.590 (0.016)
Percent body fat (%)	20.7 ± 0.9	20.6 ± 0.8	−0.09 (−0.93, 0.75)	20.6 ± 1.1	20.4 ± 1.4	−0.15 (−0.99, 0.70)	0.806 (0.003)	0.150) (0.112)	0.476 (0.029)

Note. Values are expressed as mean ± standard deviation. NTG, normoxic training group; HTG, hypoxic training group; CI, confidential interval; BMI, body mass index; FFM, free fat mass.

### Hematological Parameters

Table 3 shows the pre- and post-training hematological parameter data. There was a significant interaction in Hct (*η* ^2^ = 0.320, *p* = 0.009), EPO (*η* ^2^ = 0.246, *p* = 0.026), neutrophils (*η* ^2^ = 0.317, *p* = 0.010), and B cells (*η* ^2^ = 0.236, *p* = 0.030). In addition, there was a significant main effect within time in monocytes (*η* ^2^ = 0.715, *p* < 0.001), basophils (*η* ^2^ = 0.253, *p* = 0.024), and lymphocytes (*η* ^2^ = 0.334, *p* = 0.008). In the post-hoc analysis, EPO showed a significant increase (Cohen’s d: NTG 0.93, 95% CI: 0.00, 1.97; HTG 1.84, 95% CI: 0.76, 2.76, *p* < 0.05), and monocytes showed a significant decrease (Cohen’s d: NTG −1.20, 95% CI: −2.06, −0.23; HTG -0.93, 95% CI: −1.77, −0.01, *p* < 0.05) in the NTG and HTG. B cells were significantly increased (Cohen’s d: 1.31, 95% CI: 0.33, 2.18, *p* < 0.05) in the NTG. Hct (Cohen’s d: 0.67, 95% CI: −0.22, 1.51, *p* < 0.05) and neutrophils (Cohen’s d: 0.74, 95% CI: −0.16, 1.58, p < 0.05) significantly increased, and lymphocytes significantly decreased (Cohen’s d: −0.54, 95% CI: −1.37, 0.34, *p* < 0.05) in the HTG.

**TABLE 3:** Changes in the hematological parameters before and after 6 weeks exercise training in each environmental condition.

Variables	NTG (*n* = 10)		Cohen’s d (95% CI)	HTG (*n* = 10)		Cohen’s d (95% CI)	*p*-value (*η* ^2^)		
	Pre	Post		Pre	Post		Group	Time	Interaction
RBC (10^6^/uL)	4.5 ± 0.3	4.4 ± 0.3	−0.28 (−1.11, 0.58)	5.0 ± 0.3	5.0 ± 0.5	−0.08 (−0.92, 0.76)	0.003 (0.402)†	0.356 (0.047)	0.730 (0.007)
Hb (g/dl)	14.2 ± 0.8	14.0 ± 1.1	−0.12 (−0.96, 0.72)	14.9 ± 0.7	14.9 ± 0.6	0.02 (−0.82, 0.85)	0.031 (0.232)†	0.488 (0.027)	0.427 (0.035)
Hct (g/dl)	43.4 ± 2.3	43.1 ± 2.1	−0.13 (−0.96, 0.72)	44.5 ± 1.3	46.0 ± 2.3	0.67 (−0.22, 1.51)*	0.031 (0.232)†	0.065 (0.177)	0.009 (0.320)†
EPO (mIU/ml)	10.0 ± 1.2	12.1 ± 2.7	0.93 (0.00, 1.77)*	10.7 ± 3.3	16.4 ± 3.0	1.84 (0.76, 2.76)*	0.015 (0.285)†	0.000 (0.608)†	0.026 (0.246)†
WBC (103/uL)	5.0 ± 0.3	5.0 ± 0.5	0.15 (−0.70, 0.99)	5.2 ± 0.9	5.3 ± 1.0	0.16 (−0.68, 1.00)	0.474 (0.029)	0.107 (0.138)	0.430 (0.035)
Monocyte (%)	10.2 ± 1.9	8.0 ± 1.7	−1.20 (−2.06, −0.23)*	11.5 ± 2.8	9.0 ± 2.6	−0.93 (−1.77, −0.01)*	0.249 (0.073)	0.000 (0.745)†	0.552 (0.020)
Eosinophil (%)	2.8 ± 1.1	2.4 ± 1.0	−0.41 (−1.24, 0.46)	3.0 ± 1.6	3.1 ± 2.5	0.04 (−0.80, 0.88)	0.500 (0.026)	0.453 (0.032)	0.248 (0.073)
Neutrophil (%)	54.3 ± 5.1	57.0 ± 7.5	0.37 (−0.50, 1.20)	49.4 ± 9.2	57.4 ± 10.7	0.74 (−0.16, 1.58)*	0.543 (0.021)	0.000 (0.655)†	0.010 (0.317)†
Basophil (%)	1.1 ± 0.2	0.9 ± 0.2	−0.75 (−1.58, 0.15)	1.2 ± 0.4	1.1 ± 0.4	−0.25 (−1.08, 0.61)	0.195 (0.091)	0.024 (0.253)†	0.825 (0.003)
Lymphocyte (%)	37.9 ± 4.9	33.3 ± 8.8	−0.61 (−1.45, 0.27)	40.0 ± 4.0	35.7 ± 7.0	−0.54 (−1.37, 0.34)*	0.380 (0.043)	0.008 (0.334)†	0.914 (0.001)
B-cell (%)	11.5 ± 1.5	13.4 ± 1.5	1.31 (0.33, 2.18)*	15.2 ± 2.5	15.3 ± 2.6	0.05 (−0.79, 0.89)	0.004 (0.380)†	0.015 (0.288)†	0.030 (0.236)†
T-cell (%)	81.4 ± 6.7	80.8 ± 6.1	−0.09 (−0.92, 0.76)	74.7 ± 4.7	71.9 ± 5.9	−0.51 (−1.34, 0.36)	0.006 (0.353)†	0.081 (0.159)	0.241 (0.075)
NK cell (%)	13.7 ± 6.2	12.2 ± 5.0	−0.22 (−1.05, 0.63)	16.6 ± 1.7	19.1 ± 6.4	0.51 (−0.37, 1.34)	0.030 (0.237)†	0.611 (0.015)	0.066 (0.176)

Note. Values are expressed as mean ± standard deviation. NTG, normoxic training group; HTG, hypoxic training group; RBC, red blood cell, Hb = hemoglobin, Hct = hematocrit, EPO, erythropoietin; WBC, white blood cell; NK cell = natural killer cell; CI, confidence interval; Significant interaction or main effect: †*p* < 0.05; Significant difference between the pre- and post-tests: *p < 0.05.

### Hemodynamic Function

**FIGURE 2:**
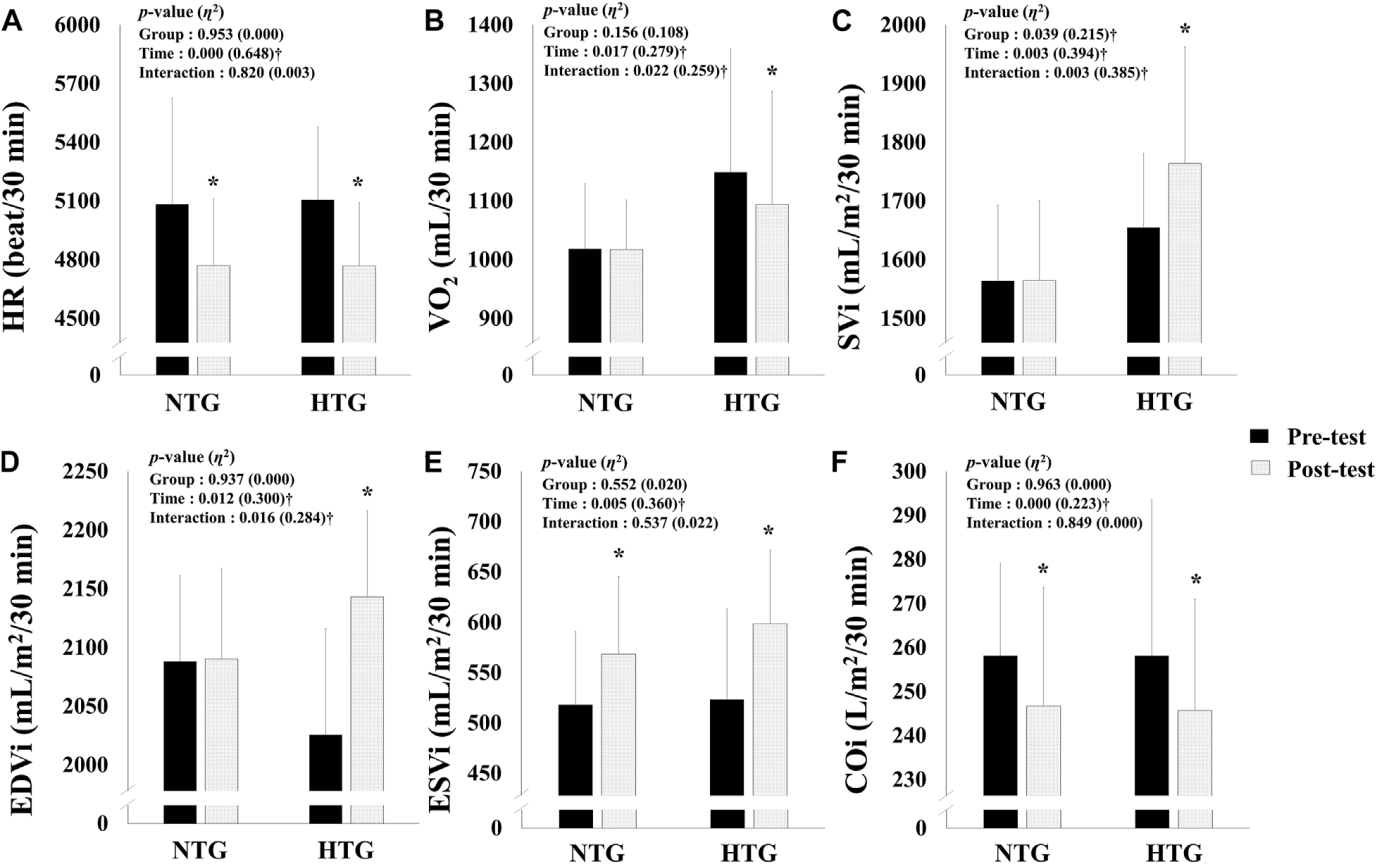
Changes in hemodynamic function parameters during 30 min of submaximal exercise before and after 6 weeks exercise training in each environmental condition. **(A)** Change in HR. **(B)** Change in VO_2_. **(C)** Change in SVi. **(D)** Change in EDVi. **(E)** Change in ESVi. **(F)** Change in COi. Note. NTG = normoxic training group, HTG = hypoxic training group, HR = heart rate, VO_2_ = oxygen uptake, SVi = stroke volume index, EDVi = end-diastolic volume index, ESVi = end-systolic volume index, COi = cardiac output index. Significant interaction or main effect: †*p* < 0.05; Significant difference between the pre- and post-tests: *p < 0.05.

Figure 2 depicts the pre- and post-training session hemodynamic function parameter data during a 30 min bout of submaximal cycle ergometer exercise corresponding to 75% HRmax. There was a significant interaction in VO_2_ (*η* ^2^ = 0.259, *p* = 0.022), SVi (*η* ^2^ = 0.385, *p* = 0.003), and EDVi (*η* ^2^ = 0.284, *p* = 0.016). In addition, there was a significant main effect of time on the HR (*η* ^2^ = 0.648, *p* < 0.001), ESVi (*η* ^2^ = 0.360, *p* = 0.005), and COi (*η* ^2^ = 0.223, *p* < 0.001). In the post-hoc analysis, HR (Cohen’s d: NTG −0.58, 95% CI: −1.41, 0.30; HTG −0.92, 95% CI: −1.76, 0.00, p < 0.05) and COi (Cohen’s d: NTG −0.33, 95% CI: −1.16, 0.53; HTG 1.84, 95% CI: −1.20, 0.49, p < 0.05) showed a significant decrease, and ESVi showed a significant increase (Cohen’s d: NTG 0.67, 95% CI: −0.22, 1.50; HTG 0.91, 95% CI: −0.01, 1.75, *p* < 0.05) in the NTG and the HTG. In addition, VO_2_ showed a significant decrease (Cohen’s d: −0.26, 95% CI: −1.09, 0.59, p < 0.05), and SVi (Cohen’s d: 0.41, 95% CI: −0.45, 1.24, p < 0.05) and EDVi (Cohen’s d: 0.78, 95% CI: −0.12, 1.62, p < 0.05) showed significant increases in the HTG.

### Endurance Exercise Performance

**FIGURE 3:**
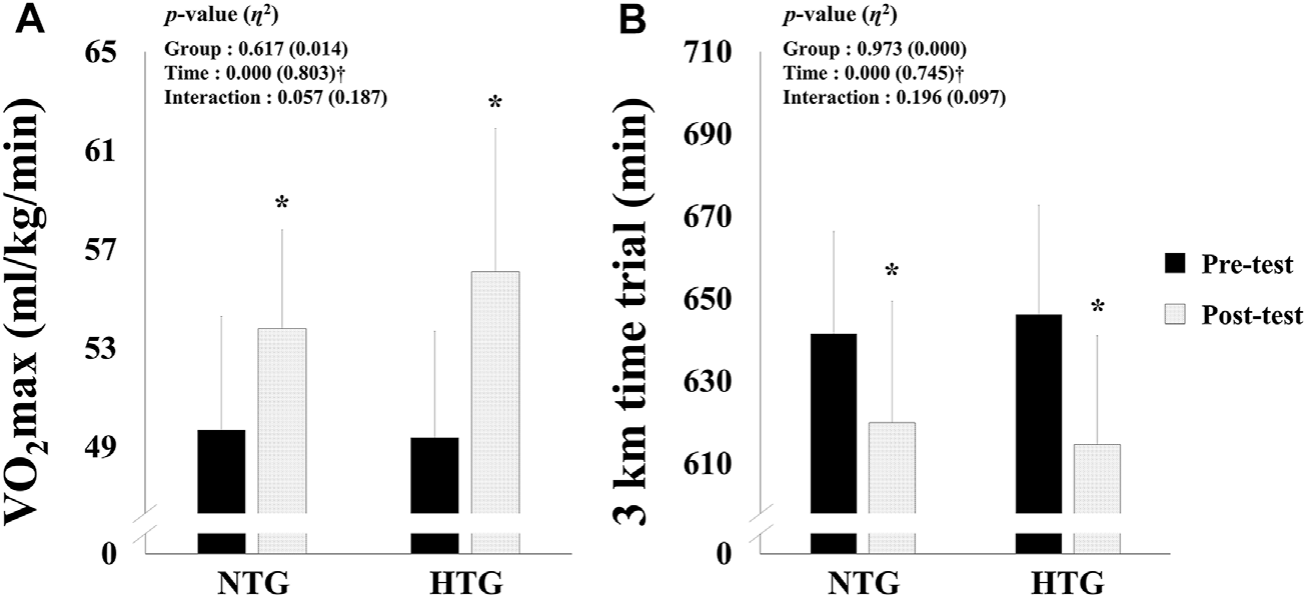
Changes in endurance exercise performance parameters before and after 6 weeks exercise training in each environmental condition. **(A)** Change in VO_2_max. **(B)** Change in 3 km time trial. Note. NTG = normoxic training group, HTG = hypoxic training group, VO_2_max = maximal oxygen uptake. Significant interaction or main effect: †*p* < 0.05; Significant difference between the pre- and post-tests: *p < 0.05.

The endurance exercise performance parameters are shown in Figure 3; no significant interaction was found. However, there was a significant main effect within time in VO_2_max (*η* ^2^ = 0.803, *p* < 0.001) and 3000 m time trial records (*η* ^2^ = 0.754, *p* < 0.001). In the post-hoc analysis, VO_2_max (Cohen’s d: NTG 0.94, 95% CI: 0.02, 1.79; HTG 1.15, 95% CI: 0.19, 2.00, *p* < 0.05) and 3000 m time trial records (Cohen’s d: NTG -0.78, 95% CI: −1.61, 0.12; HTG −1.19, 95% CI: −2.05, −0.23, *p* < 0.05) were significantly improved in both groups, and the HTG showed a tendency to improve VO_2_max (NTG: 8.2 *vs*. 13.6%) and 3000 time trial records (3.4 *vs.* 4.9%) more than the NTG.

## Discussion

Our study demonstrated that the IHT regimen is an effective training method for enhancing endurance exercise performance due to its effects on hematological parameters regarding oxygen transporting capacity and hemodynamic function. Hematological parameters regarding immune system showed a normal range before and after training, which indicates the safety of hypoxic training.

### Hematological Parameters

The hematological parameters related to oxygen transport capacity show that hypoxic exposure with an effective hypoxia dose (e.g., appropriate exposure level, duration, and time) stimulates erythropoiesis (e.g., an increase in Hb mass and RBC count) and improves the oxygen transport capacity of the blood, thus leading to enhanced VO_2_max and endurance exercise performance in individuals under normoxia (Levine and Stray-Gundersen, 1997; Dehnert et al., 2002; Gore et al., 2006; Robach et al., 2006; Schmitt et al., 2006; Gore et al., 2013). Most previous studies reporting increased red cell volume, red cell mass, and Hb mass caused by erythropoiesis following hypoxic training have utilized the living-high training-low (LHTL) regimen (Levine and Stray-Gundersen, 1997; Robach et al., 2006; Wehrlin et al., 2006). Several previous studies using the LHTL regimen reported mean increases of 5–9% in red cell volume and >8% in Hb concentration (Levine and Stray-Gundersen, 1997; Rusko et al., 1999; Hahn et al., 2001; Stray-Gundersen et al., 2001). In a previous study (Sinex and Chapman, 2015), an LHTL regime residing at 2000–2,500 m or lower for 3–4 weeks with over 12 h of continuous altitude exposure per day appeared to be sufficient to activate an EPO response and RBC production.

However, our study examined hematological parameters related to oxygen-transporting capacity using the IHT regime instead of the LHTL regime in amateur Korean female runners. In general, the IHT regime does not have sufficient hypoxic stimulation due to shorter hypoxic exposure (e.g., two–five sessions per week of <3 h during four to 6 weeks) and thus does not induce hematological changes related to oxygen transporting capacity such as erythropoiesis (McLean et al., 2014; Park et al., 2018; Park et al., 2020). Regarding the IHT regime and hematological changes, Ramos-Campo et al. analyzed modifications in hematological and aerobic performance parameters after a 7-weeks intermittent hypoxia training (IHT) program (Ramos-Campo et al., 2015). All participants (*n* = 18) were divided into an intermittent hypoxia training group (IHTG) that conducted a normoxic training plus an intermittent training under hypoxia (inspired oxygen fraction; FiO_2_ = 14.5–15%) and a control group (CG) that performed only a normoxic training. They reported higher Hb and RBC values in the IHTG than in CG but did not clearly elucidate the mechanism for erythropoiesis following IHT. Kasperska and Zembon-Lacny evaluated changes in EPO levels and hematological parameters in wrestlers after intermittent hypoxic exposure (IHE) (Kasperska and Zembron-Lacny, 2020). Twelve wrestlers were assigned to a hypoxic group (training combined with IHE, *n* = 6) and control group (training, *n* = 6), and the hypoxic group was exposed to hypoxia (FiO_2_ = 12–13.5%) for 10 days, with one day off after 6 days, once a day for about an hour. They concluded that 10-days IHE with wrestling training improves the oxygen transporting capacity by the release of EPO and reticulocytes in circulation. Camacho-Cardenosa et al. verified the effects of a new dose of maximal-intensity interval training in hypoxia on hematological parameters in active twenty-four university student volunteers (Camacho-Cardenosa et al., 2017). The participants divided into a control group, a normoxia group, and a hypoxic group, and the eight training sessions consisted of 2 sets of 5 repeated sprints of 10 s with a recovery of 20 s between sprints and a recovery period of 10 min between sets. They reported that hypoxic group showed a significantly higher Hct and Hb concentration. Camacho-Cardenosa et al. evaluated changes in Hct and Hb in healthy participants following repeated-sprint training in hypoxia and they reported that repeated sprint training in hypoxia significantly increased Hct and Hb concentration (Camacho-Cardenosa et al., 2021).

In the present study, we confirmed a significantly increased EPO concentration (53.3%) and Hct (3.4%) in the HTG using the IHT regimen. EPO is a glycoprotein hormone secreted by the kidneys and is the most influential factor in hematological changes that rapidly increase in the secretion phase in response to exposure to hypoxia (Park et al., 2016; Kasperska and Zembron-Lacny, 2020). Exposure to hypoxia sharply increases EPO secretion and reaches maximal secretion within about 48–72 h, and new erythropoiesis by EPO stimulation takes approximately 5 days under persistent hypoxia (Ge et al., 2002; Gore et al., 2006). Exercise training under hypoxia is effective in improving the oxygen transport capacity of the blood because it increases the plasma volume and red blood cell production by EPO, and this effect lasts for approximately 16 days after exposure to hypoxia (Gore et al., 2013; Płoszczyca et al., 2018). However, the present study did not show an increase in RBC and Hb concentrations, which confirms that our IHT showed an increase in EPO but not a hypoxic dose sufficient to induce erythropoiesis. In addition, the increase in Hct is understood to be a result of insufficient hydration to compensate for body water loss during the 6-weeks training period, probably because RBC and Hb concentrations did not change (Park et al., 2020).

Regarding hematological parameters related to immune system, exercise training under hypoxia acts as a stressor and thus induces changes in the nervous and endocrine systems, consequently affecting immune-related parameters (Mazzeo et al., 2001; Mazzeo, 2008; Jung et al., 2020; Park et al., 2020). It is important to confirm its safety, because alterations in immune system induced by hypoxic exposure can potentially adversely affect health (Wilber, 2007). In previous studies related to hypoxic training and immune system, Park et al. reported that 2 weeks of continuous and interval training under hypoxia (596–526 mmHg, 2000–3000 m simulated altitude) in Korean national cycling athletes with disabilities resulted in significantly decreased leukocytes and natural killer cells and increased eosinophils, B cells, and T cells; however, the effect of hypoxic training on immune system is unclear because all indicators are in the normal range (Park et al., 2020). Jung et al. evaluated the effects of 6-weeks IHT (526 mmHg, 3000 m simulated altitude) on immune system in middle- and long-distance runners (Jung et al., 2020). They reported that the HTG showed a more significant increase in the WBC and neutrophil counts and a significant decrease in the monocyte count than the NTG; however, all immune system parameters were stable within the normal range before and after training. While the hypoxic training regime was different from that in the present study, Brugniaux et al. investigated the effect of 13–18 days of the LHTL regime (training at 1,200 m and sleeping at 3000–3500 m) on leukocyte count in 41 athletes from 3 federations (cross-country skiers, *n* = 11; swimmers, *n* = 18; runners, *n* = 12) and reported that leukocyte count was not affected except at 3,500 m (Brugniaux et al., 2006). Tiollier et al. investigated the effect of the LHTL regime (training at 1,200 m and residing at 2,500–3500 m) for 18 days on secretory immunoglobulin A (sIgA) levels in 6 female and 5 male elite cross-country skiers (Tiollier et al., 2005). They reported a downward trend in sIgA concentrations over the study period, which was significant in the LHTL group. These results strongly suggested a cumulative negative effect of LHTL training on sIgA levels.

In the present study, we observed a significant increase in neutrophils and decreased monocyte and lymphocyte counts in the HTG, and B cells were significantly increased in the NTG. However, hematological parameters related to immune system were altered after 6 weeks of IHT and were clinically within the normal range. These findings suggest that unlike the LHTL regime wherein there was exposure to hypoxia for a long time, the IHT regime did not negatively affect immune system, which is consistent with the results of a previous study (Brugniaux et al., 2006; Jung et al., 2020; Park et al., 2020).

### Hemodynamic Function

Hemodynamic function represents the dynamics of blood flow for oxygen delivery to the tissue under systemic conditions, and the hemodynamic system continuously monitors and adjusts the state of the body and environment changes during exercise (Green et al., 2000; Park et al., 2018). Excise training under hypoxia enhances endurance exercise performance by increasing oxygen transport and utilization efficiency and energy availability, which consequently improves the invigoration of the parasympathetic nervous system *via* the activation of *ß*-adrenergic receptors in the cardiac muscles and efficiently changes cardiac function by increasing venous return (Park et al., 2019). In other words, endurance exercise performance is strongly related to hemodynamic function, which is a parameter of oxygen transport and utilization capacity (Park et al., 2018).

In addition, previous studies have reported that exercise training under hypoxia enhances endurance exercise performance by increasing mitochondrial density, capillary density, oxidative enzyme activity, motor unit activity, glycolysis enzyme activity, glucose delivery capacity, lactate tolerance, acid-base balance regulation, and oxygen utilization capacity (Geiser et al., 2001; Hamlin et al., 2010; Czuba et al., 2011; Park et al., 2016). In other words, IHT may enhance endurance exercise performance by inducing various physiological, biochemical, and structural adaptive alterations in cardiac and skeletal muscles that favor oxidative energy production and can enhance non-hematological parameters (e.g., exercise economy, acid-base balance, and metabolic response during exercise), ultimately leading to improved oxygen delivery and utilization capacity (Hamlin et al., 2010; Faiss et al., 2013; Girard et al., 2017; Park et al., 2018; Jung et al., 2020). Exercise economy enhances endurance exercise performance, which means improved oxygen inflow rate into skeletal muscle tissue and oxygen utilization capacity in the mitochondria (Green et al., 2000; Saunders et al., 2004). Previous studies have reported that physiological adaptation to high-intensity hypoxic training improves exercise economy and endurance exercise performance by improving adenosine triphosphate (ATP) resynthesis (e.g., per 1 mol of oxygen) and decreasing ATP levels at a given exercise intensity (e.g., speed and workload) performance (Green et al., 2000; Gore et al., 2001; Truijens et al., 2003; Park et al., 2018).

In the present study, VO_2_ significantly decreased, and SVi and EDVi increased in the HTG. Additionally, HR and COi significantly decreased, and ESVi increased in both groups; however, the HTG showed a greater tendency to improve than the NTG. These results are consistent with those of previous studies, and the changes in hemodynamic function and exercise economy during submaximal exercise indicate that the ability of cardiac system to deliver blood (e.g., oxygen and nutrients) from the heart to the skeletal muscle tissue has changed efficiently, and the ability to use the oxygen delivered to the muscle tissue has improved (Green et al., 2000; Gore et al., 2001; Katayama et al., 2004; Saunders et al., 2004; Park et al., 2019).

### Endurance Exercise Performance

Regarding endurance exercise performance, most generally, oxygen transport system efficiency is most often evaluated by VO_2_max (Czuba et al., 2011; Park and Lim, 2017). IHT may enhance endurance exercise performance under normoxia through various physiological, biochemical, and structural adaptive changes (Geiser et al., 2001; Hamlin et al., 2010; Faiss et al., 2013). Researchers have confirmed that high-intensity training under hypoxia enhances VO_2_max, anaerobic threshold, maximal exercise intensity, and sports event-specific performance (Roels et al., 2005; Dufour et al., 2006; Czuba et al., 2011; Park et al., 2018). However, several previous studies failed to demonstrate an enhancement in endurance exercise performance (Vallier et al., 1996; Hendriksen and Meeuwsen, 2003). These conflicting results may be due to methodological differences in hypoxic training, such as participants’ training status, training type and intensity, dose of hypoxic stimulus, and time point of endurance performance measurement following the IHT procedure (Hamlin et al., 2010; Park et al., 2018). In particular, exercise type and intensity may be key factors in mediating the change in endurance exercise performance by IHT (Millet et al., 2010; Faiss et al., 2013; McLean et al., 2014). Hypoxic training using the high-intensity and anaerobic interval method has been shown to enhance endurance exercise performance by inducing peripheral adaptations related to various metabolic functions (Sinex and Chapman, 2015; Park et al., 2018).

In the present study, amateur female runners performed the high-intensity hypoxic training on a treadmill composed of 20 min warm-up with 60% HRmax, 60 min high-intensity of interval training under hypoxia with 90–95% HRmax, and 20 min cool-down with 60% HRmax. As a result, endurance exercise performance (e.g., VO_2_max and 3000 m time trial) was enhanced in both groups; however, we confirmed that HTG showed a greater tendency to improve VO_2_max (HTG: 13.6% *vs*. NTG: 8.2%) and 3000 m time trial (HTG: 4.9% *vs*. NTG: 3.4%) than NTG. Our results are consistent with those of previous studies that demonstrated that hypoxic training may enhance endurance exercise performance when sufficient intensity and volume of exercise training are provided based on high-intensity interval training and repeated sprint training (Millet et al., 2010; Faiss et al., 2013; McLean et al., 2014; Park et al., 2018; Camacho-Cardenosa et al., 2020).

### Limitations

The present study has the following limitations: 1) the participants of our study were amateur female runners, not elite athletes, and there are limitations in the interpretation of the effect of enhancing endurance exercise performance following IHT; 2) individual responses to hypoxia, that is, exercise intensity settings according to hypoxic ventilatory response, were not performed in our study; 3) except for the exercise training performed in our study, other physical activities were restricted, but we did not investigate daily activity, dietary intake, and hydration of participants during the training period; 4) measurements of menstrual cycle and sex hormones were not performed despite the participants being female; and 5) The normality of distribution of all outcome parameters was verified using the Shapiro–Wilk W-test prior to the parametric tests, however the small sample size was a limitation to elicit the effectiveness of IHT on hematological parameters, hemodynamic function, and endurance exercise performance in amateur female runners in Korea.

## Conclusions

In conclusion, the present study verified that the IHT was effective in enhancing endurance exercise performance with improved hemodynamic function, and hematological parameters regarding immune system (e.g., WBC, monocyte, eosinophil, neutrophil, basophil, lymphocyte, B-cell, T-cell, and NK cell) showed a normal range before and after training and were not negatively affected.

## Data Availability

The raw data supporting the conclusion of this article will be made available by the authors, without undue reservation.
